# Drug delivery vectors based on filamentous bacteriophages and phage-mimetic nanoparticles

**DOI:** 10.1080/10717544.2017.1410259

**Published:** 2017-12-01

**Authors:** Zhigang Ju, Wei Sun

**Affiliations:** a Medicine College, Guiyang University of Chinese Medicine, Huaxi university town, Guiyang City, Guizhou Province, China;; b Key Laboratory of Plant Physiology and Development Regulation, College of Life Science, Guizhou Normal University, Huaxi university town, Guiyang City, Guizhou Province, China

**Keywords:** Filamentous phage, phage display, nanoparticles, targeting, drug delivery

## Abstract

With the development of nanomedicine, a mass of nanocarriers have been exploited and utilized for targeted drug delivery, including liposomes, polymers, nanoparticles, viruses, and stem cells. Due to huge surface bearing capacity and flexible genetic engineering property, filamentous bacteriophage and phage-mimetic nanoparticles are attracting more and more attentions. As a rod-like bio-nanofiber without tropism to mammalian cells, filamentous phage can be easily loaded with drugs and directly delivered to the lesion location. In particular, chemical drugs can be conjugated on phage surface by chemical modification, and gene drugs can also be inserted into the genome of phage by recombinant DNA technology. Meanwhile, specific peptides/proteins displayed on the phage surface are able to conjugate with nanoparticles which will endow them specific-targeting and huge drug-loading capacity. Additionally, phage peptides/proteins can directly self-assemble into phage-mimetic nanoparticles which may be applied for self-navigating drug delivery nanovehicles. In this review, we summarize the production of phage particles, the identification of targeting peptides, and the recent applications of filamentous bacteriophages as well as their protein/peptide for targeting drug delivery *in vitro* and *in vivo*. The improvement of our understanding of filamentous bacteriophage and phage-mimetic nanoparticles will supply new tools for biotechnological approaches.

## 1. Introduction

Since the presentation of “side chain theory of immunity” and “magic bullet concept” by Paul Ehrlich more than 100 years ago (Strebhardt & Ullrich, 2008; Bertrand et al., 2014; Vigevani & Valcárcel, 2014), a vast variety of novel drugs have been discovered. With the development of nanomedicine, more and more carriers have also been developed for targeted delivery of these new drugs to exert therapeutic effects, such as liposomes, dendrimers, polymers, micelles, virus-like particles, and even stem cells (Peer et al., 2007; Blanco et al., 2011; Wang et al., 2012; Cao et al., 2014). Normally, an ideal delivery vector should possess several special properties, such as good biocompatibility, proper hydrophilicity, targeting specificity, low toxicity, high uptake efficiency, and so on (Ma et al., 2012). However, all existing delivery systems have some inherent shortcomings more or less. For example, liposomes, which have been approved by US Food and Drug Administration (FDA) and widely used in clinics (Noble et al., 2014), are easily degraded *in vivo* and their large size (>100 nm) will hinder the penetration and diffusion (Longmire et al., 2011; Wen et al., 2013).

To date, safety and efficiency are two main evaluation criteria during drug delivery (Ryvolova et al., 2013), and how to improve their performance has been a hot topic of modern medical research. Although many therapeutic agents have been proposed for disease treatment, the therapeutic effect is still less than satisfactory. This phenomenon is mainly caused by the following several aspects: drugs are degraded before reaching the lesion sites; low target-specificity results in severe side effects; the quantity of drugs delivered into the cells is not sufficient for effective exertion and so on. Among all these factors, targeting is one of the key elements. Lately, as the excellent specificity, antibody has been proposed and applied for targeting delivery. However, because of “binding site barrier” and rapid clearance, antibody is not the best choice as a targeting motif in a targeted delivery system (Osdol et al., 1991). Subsequently, depending on the properties of peptide, such as small size, easy synthesis and typically non-immunogenicity (Hart et al., 1995; Bray, 2003; Ruoslahti, 2012; Bakhshinejad et al., 2014), it was widely used as targeting specific molecule from single target to complex multicomponent machinery (Dobbelstein & Moll, 2014). But the chemical synthesis of peptide is much more cost-effective than the production of antibodies. In case the peptides can be expressed and displayed directly on the surface of nanocarrier, such as filamentous phage, the cost of peptides will be negligible, and this is very meaningful for the development of novel drug delivery nanocarriers.

**Figure 1.**
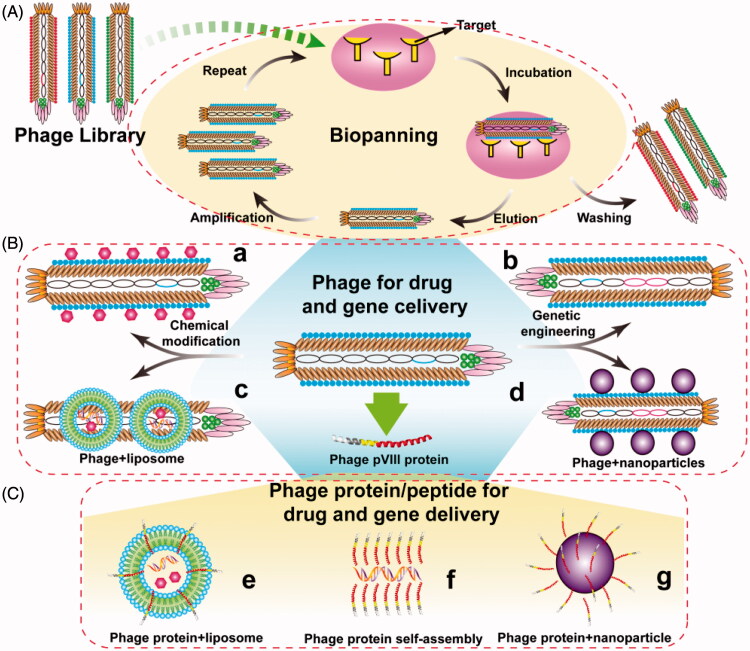
General concept of using phage for drug and gene delivery. (A) Identification of target-recognizing peptide through bio-screening. A phage library is mixed with immobilized targets and incubated for a proper time. Unbound phages are then washed away with a washing buffer. Bound phages are eluted with an elution buffer, and amplified using medium containing preincubated *E.coli* bacteria and then acted as a new input library for next round bio-screening. After 3 ∼ 5 rounds, the selected phage clones are identified. (B) The paradigm of drug and gene delivery using phage particles. Phage can be chemically modified and/or genetically engineered to load drugs (a) and carry foreign genes (b), respectively. Phage can also be incorporated with other nanometer carriers for drug and gene delivery, such as liposomes (c) and nanoparticles (d). (C) The paradigm of drug and gene delivery using phage-borne proteins. Wild type or fused phage proteins can be inserted into liposomes (e) and polymer nanoparticles (g) to form phage-mimetic complexes, and even self-assembly into nanophage (f) to deliver drug and gene.

Bacteriophage (generally called phage), a kind of virus, was discovered by Frederick Twort and F´elix d’H´erelle in 1915 and 1917, respectively (Kaur et al., 2012). Compared with other viruses, one of the significant advantages of phage is their non-infectivity to mammalian cells. Besides, small genome, simple structure, and easy engineering are also the major properties of phage. So biologists, chemists, materials scientists, and medical scientists have paid more and more attentions to the phage. Since the technology of phage display was first elaborated by George P. Smith (1985) via inserting a fragment of *EcoR* I endonuclease on the pIII position of filamentous phage f1, different phage display systems have been exploited and applied, including phage vector-based display system and phagemid vector-based display system. Even each system has its own advantages and disadvantages, these two systems can allow small peptides to be displayed on the surface of phage in a manner of single or multivalent display. Based on phage display technology, a phage library has been constructed and used for selection of targeting phages or peptides through biopanning. The detailed process of biopanning is showed in Figure 1(A). The selected phages or peptides can be used to develop many new functional phages or phage-mimetic particles for a variety of applications (Figure 1), such as cell-targeting, tumor-homing and cell-penetrating, etc.

Until now, phages have been widely used in immunogenic vaccine delivery (Cruz et al., 1988; Henry et al., 2015), materials synthesis (Mao et al., 2003; Mao et al., 2004; Lee et al., 2009b; Qiu & Mao, 2010; Mao et al., 2012; Huang et al., 2015), cell growth, and differentiation (Merzlyak et al., 2009; Zhu et al., 2011; Qiu et al., 2013; Wang et al., 2013; Wang et al., 2014; Kim et al., 2017), molecular imaging (Deutscher, 2010; Carrico et al., 2012; Ghosh et al., 2012; Ma et al., 2017), and battery materials (Nam et al., 2006; Oh et al., 2014; Mohan & Weiss, 2016). One of the most important applications of phage is its use as the drug and gene delivery carrier (Figure 1(B,C)). For chemical drug delivery, filamentous phages can be loaded with a great deal of chemical drugs and have superior pharmacokinetic as well as delivery efficiency comparing with spherical nanoparticles (Lee et al., 2009a; Chauhan et al., 2011; Shukla et al., 2013). For gene delivery, foreign genes can be able to insert into the genome of phage by recombinant DNA technology, and can also be loaded by phage-mimetic nanoparticles through chemical or physical methods. Previous studies have identified that the single-strand genome of fd can be converted into double-stranded DNA in mammalian cells (Bakhshinejad et al., 2014). Most importantly, phage particles can be modified with targeting or internalizing peptides by phage display, which is a significant profile different from any other gene and drug delivery system. Moreover, with this unique characteristic, phage can also combine with other nanometer carriers to produce phage-mimetic nanoparticles and exploit plenty of new delivery systems (Figure 1(B)).

So far, there are a few review articles on phage display (Smith & Petrenko, 1997; Kehoe & Kay, 2005; Sergeeva et al., 2006; Hamzeh-Mivehroud et al., 2013), phage library (Gray & Brown, 2014), phage for imaging (Deutscher, 2010), phage self-assembly structures (Moona et al., 2015), nanomaterials for gene, and drug delivery (Biju, 2014). However, to our best knowledge, there is no review on the use of phage in gene and drug delivery. So in this review, we summarize the production of phage particles and the identification of targeting peptides using for drug delivery. Meanwhile, we also discuss the recent applications of phages and phage-mimetic nanoparticles for *in vitro* and *in vivo* drug delivery.

## 2. Mass production of phage nanofibers by infecting bacteria

Filamentous bacteriophage is a kind of virus that can only infects *E.coli* containing F pilus. The most studied filamentous bacteriophages are f1, fd, and M13, which contain a single-strand DNA (ssDNA) genome encapsulated by major coat proteins (pVIII) forming the backbone of filamentous bacteriophage, and minor coat proteins at two ends, one end composed of pIII and pVI, the other end composed of pVII and pIX.

Phages are divided into temperate and virulent phage on the basis of lifestyle. Virulent phage will initial the lysis of host cells. On the contrary, filamentous bacteriophages as temperate phages do not lyse host, moreover, it can protect the host from infection by other phages. The detailed process of filamentous phage infection is demonstrated as follows: At the beginning, phages absorb to the surface of host cells by interaction of pIII and F pilus, and the phage genome is injected into the cell with coat proteins staying outside. Subsequently, single-strand DNA (ssDNA) replicates into replicative form (RF) via a rolling circle mechanism, and new coat proteins are synthesized to assemble into phage particle by taking over host metabolism and molecular mechanism. Afterwards, pV binds to ssDNA and form a rod-like structure. Finally, with the help of pI, pVII, and pIX, ssDNA anchors to the inner membrane, and pVIII self-assembly on the surface of ssDNA. Then a whole phage is successfully assembled and released from the cell.

## 3. Identification of targeting using phage libraries

Since the first proposition of phage display by George P. Smith, this technology attracted more and more attentions. By genetic engineering of phage genome and assembly of phage proteins, foreign gene sequences which encode small peptides with specific targeting ability can be displayed on the surface of phage particles. The most commonly used coat proteins for phage display are pVIII and pIII (Wang & Yu, 2004; Kehoe & Kay, 2005). There are ∼2700 copies of major coat proteins pVIII with α-helical architecture (about 20°) arranged tightly along the phage particle. To avoid incorrect assembly, only short peptides (less than 10 amino acids) are allowed to display on every copy of pVIII. Actually, only ∼5 copies of pIII are responsible for infection and assembly termination. In addition, pVI (Hufton et al., 1999), pVII (Kwaśnikowski et al., 2005), and pIX (Gao et al., 2002b) can also applied for phage display.

Based on phage display technology, various phage display libraries have been constructed, such as random peptide library, phage antibody library and phage protein library, by using Kunkel mutagenesis, PCR reaction and ligation, codon sets reduction, and incorporation of unnatural amino acids. Phage library contains a reservoir of peptides that can be used for selecting versatile ligands in biomedical area, for example cell targeting drug carriers, directed location of gene delivery vectors, and targeting and tissue penetration of nanoparticles (Ruoslahti, 2012). Furthermore, the selecting peptides can also be used to overcome the obstacles in the process of drug delivery, including specific cell binding and internalization, endosome escape, and nuclei location (Han et al., 2016; Wang et al., 2016; Staquicini et al., 2017).

### 3.1 Cell-binding peptide

Originally, phage library was only used for selection of a given known protein or antibody solution (Devlin et al., 1990; Scott & Smith, 1990; Lam et al., 1991). But now, from biomolecules to inorganic nanoparticles, from known molecules to unknown targets, from *in vitro* to *in vivo*, phage library has already been applied to select all sorts of targets. One of its significant applications is to screen peptides that can bind with cell surface, named cell-binding peptides. On the surface of a cell, there are a large number of specific macromolecules, such as integrin (Hart et al., 1994), cadherin (Devemy & Blaschuk, 2009), HER2 (Houimel et al., 2001), EGFR (Li et al., 2005) and so on. The subtle differences of these molecules can be discriminated by small specific ligands which can be captured through bio-screening of phage libraries. For example, many targeting peptides bind to a specific antigen of cancer cells have been obtained using phage libraries and reviewed somewhere else (Sergeeva et al., 2006; Gray & Brown, 2014). Otherwise, the whole cells can also be immediately utilized for screening with phage library to obtain small peptides that bind with unknown specific targets of cells.

### 3.2 Cell-penetrating peptide

Many targeting molecules, named homing peptides (HPs), only help nanocarriers deliver their attached cargoes onto the surface of cell without penetrating it (Svensen et al., 2012). However, most gene and drug delivery systems need to penetrate into cells, or escape from endosomes and lysosomes, or even translocate into nucleus. So a new kind of peptide, named cell-penetrating peptide (CPP), has been discovered and verified. This peptide can be displayed on the surface of phages and phage-like particles which can help them internalize/penetrate into cells through endocytosis receptor-mediated endocytosis or receptor-independent endocytosis (Madani et al., 2011; Milletti, 2012). Since the trans activating transcription (Tat) protein of HIV-1 reported (Frankel & Pabo, 1988; Green & Loewenstein, 1988), more and more CPPs are discovered and used for translocation of therapeutic cargoes, including oligonucleotides, proteins and nanoparticles (Heitz et al., 2009; Milletti, 2012; Ruoslahti, 2012). Till now, lots of CPPs have been identified through bio-screening of phage libraries, such as HN-1 (Hong & Clayman, 2000), pep-7 (Gao et al., 2002a), 439a, 435 b (Kamada et al., 2007) and so on.

For internalizing and penetrating of phage and phage-like nanoparticles, many mechanisms of endocytosis have been proposed. In particular case, due to its own profiles of a delivery system, the special mechanism of endocytosis needs to be identified. In other words, a specific CPP only for a specific endocytic pathway should be selected and defined. For instance, through combination of phage display bio-screening and endocytic selection, two H1299 non-small cell lung cancer CPPs were identified and proved with two different mechanism of endocytosis (McGuire et al., 2014; Umlauf et al., 2014). This is the first report on the selection of endocytosis-specific peptide through phage library. While CPPs can also help therapeutic cargoes escape from endosomes and avoid degradating by lysosomes, which is crucial for gene delivery. Because, a majority of phage particles are sequestrated by endo-lysosomal degradative pathway (Stoneham et al., 2012). In addition, significant increase of gene delivery efficiency has been achieved through disruption of endosome and lysosome by virtue of lysosomotropic chemical agents and fusion or penetration of endosome membrane (Marsh & Helenius, 2006).

### 3.3 Nuclear location peptide

After escaping from endosome and lysosome, drugs will be released as proposed. But for gene delivery, nuclear envelope is the biggest barrier, which needs to be translocated. Luckily, nuclear localization signal (NLS) can be recognized by the nuclear transport proteins and help genes arrive at the nucleus. It has been reported that NLSs exist in the terminal proteins (TPs) of many bacteriophages, such as Ф29, Nf, PRD1, Bam35, Cp-1 and YS61 (Redrejo-Rodríguez et al., 2012; Redrejo-Rodríguez et al., 2013; Redrejo-Rodríguez & Salas, 2014). At present, no NLSs have been found in filamentous bacteriophage. And this may be the reason of low transduction efficiency (4%∼10%) in mammalian cells when transduced with filamentous bacteriophage (Poul & Marks, 1999; Larocca et al., 2001). So some new strategies have been proposed. For example, the inverted terminal repeats (ITRs) in adeno-associated virus were inserted into filamentous bacteriophages for improving expression efficiency of foreign genes (Hajitou et al., 2007).

In a word, drug and gene delivery is a sophisticated process, and most previous studies only endowed phage targeting ability to bind with cell surface or internalize into the cell. However, after entering into cells, phage particles need to escape from endosomes or lysosomes and arrive at nucleus. But the detailed mechanism is still unknown. Interestingly, phage library offers a reservoir of different peptides can exert different effects in drug and gene delivery through biopanning *in vitro* and *in vivo* (Krag et al., 2006).

## 4. Filamentous phage-mediated delivery

As demonstrated above, phage as a delivery system can be applied for treating different diseases, such as bacterial infection (Yacoby et al., 2006; Gravitz, 2012; Qadir, 2015; Bardy et al., 2016; Pires et al., 2016), tumor (Gandra et al., 2013a; Bakhshinejad et al., 2014; Bedi et al., 2014; Yata et al., 2014; Gross et al., 2016; Hou & Meng, 2017), Alzheimer’s disease (Frenkel & Solomon, 2002; Munke et al., 2017) and so on.

### 4.1 Targeted gene delivery by filamentous phage

With the enhancement of tolerance to antibiotics, phage therapy has demonstrated to be a new renaissance for antimicrobial therapy. As well known, lytic phage can infect bacteria and result in cell death by lysis, but at the same time, the released endotoxin will produce severe side effects (Slopek et al., 1982). Hence, non-lytic phage was engineered to deliver lethal genes and used for antibacterial therapy (Hagens & Bläsi, 2003; Westwater et al., 2003; Hagens et al., 2004). Beyond that, the genetically engineered M13 phage can also transfers genes into bacteria and renders them more sensitive to antibiotics (Lu & Collins, 2009; Edgar et al., 2012).

Originally, lambda phage was used to transducer mammalian cells for tumor therapy as early as 1975, but failed (Horst et al., 1975). Later, recombinant f1 phage containing urokinase type-plasminogen activator (u-PA) was used for transfection of simian COS-7 cells with the help of DEAE dextran (Yokoyama-Kobayashi & Kato, 1993) or lipopolyamine (Yokoyama-Kobayashi & Kato, 1994), leading to an increased efficiency of DNA transfection. Later, Andrew Baird (2011) proposed a detailed protocol about how to transfer mammalian cells using filamentous bacteriophage. However, all these phage vectors lack targeting capacity.

With the advent of “internalizing phages”, phage-mediated gene delivery is further developed. RGD peptide fused with phage proteins can mediate the internalization of DNA into cells (Hart et al., 1994). The first report about gene transfer of mammalian cells (COS-1 cells) by genetically targeted filamentous phage was published in 1999 by David Larroca et al. (1999), in which the phage was engineered with FGF2 and GFP as pIII fusion protein and report protein respectively. In addition, Poul and Marks (1999) further confirmed the feasibility of phage particles for gene transfer to SKBR3 breast tumor cells using the multivalently displayed anti-ErbB2 scFv as a target and GFP as a reporter, but the infection efficiency was very low. Soon after, David Larroca (2001) applied a multivalent phagemid vector for targeted delivery of GFP into PC-3 cells and improved the transduction up to 10%, which is still much lower than the traditional methods.

As well known, eukaryotic virus can provide superior gene delivery and transduction, despite their native tropism to mammalian cells. If the excellent properties can combine with phage particles, the gene transfer efficiency by target phage will definitely be improved. So a new system containing the engineered RGD-targeting filamentous phage and the cis-elements of adeno-associated virus (AAV) was introduced (Hajitou et al., 2006; Hajitou et al., 2007). This novel AAV/phage (AAVP) chimers were applied to enhance the delivery and expression of various genes, such as TNF-α for antivascular therapy using M21 cells (Tandle et al., 2009) and HSVtk suicide gene for cancer using SVEC4-10-transformed murine small vessel endothelial cells (Trepel et al., 2009). But the transduce efficiency is still low, ranging from 10% to 20% in different cancer cells (Trepel et al., 2009). With the purpose of advancing the efficiency of AAVP system, the stress-inducible Grp78 promoter was introduced instead of CMV promoter. Then after treating with histone deacetylation inhibitor and DNA methylation inhibitor, the efficiency of AAVP-mediated gene transfer was significantly improved (Kia et al., 2013). Later, a M13_RGD8_-AAV_GFP_ hybrid phage was constructed using for gene delivery, and related analysis revealed that this hybrid phage could achieve selective delivery and induce GFP expression into MC3T3 cells (Yoo et al., 2016). Moreover, in order to improve anticancer safety therapy of AAVP system, this system was further modified by combining AAVP together with natural dietary genistein which had anticancer activity, and the results showed increased cell killing. (Tsafa et al., 2016).

In addition, the electrostatic repulsion between phage particles and cell surface as well as buffering capacity of phage may also affect the transduction efficacy. Therefore, a novel hybrid phage/polymer complex was developed and used for gene delivery through combining recombinant phage and cationic polymers (PDL and DEAE-DEX) (Yata et al., 2014). And the transduction efficiency of this novel hybrid phage/polymer was increased compared with no polymer modified phage. The researchers believed that this cationic polymer can help phage escape from endosomes. Hence it is feasible to deliver genes into mammalian cells using phage particles, and what we need to do is how to improve its transduction efficiency.

### 4.2 Targeted drug delivery by filamentous phage

Since 1996, monoclonal antibodies (mAbs) as therapeutics have already been reported and some of them have been approved by FDA (Reichert, 2008; Sievers & Senter, 2013). Except for targeting to the antigen on the surface of cells, mAbs or their fragments can also be displayed on a phage and act as new therapeutics for diagnosis and treatment of diseases. More importantly, phage possesses the ability to load antibodies and preserve their biological activities.

Apart from antibody, a substantial number of drugs are emerged as therapeutics for human diseases, such as antibiotics (chloramphenicol), anticancer drugs (doxorubicin), toxins, PDT agents (photosensitizer), radionuclides, cytokines, and so on. Due to higher toxicity to host and less sufficient of drug quantities, more and more drugs have been excluded from therapeutics. While, as a robust scaffold, filamentous phage can be applied to overcome these problems by chemical modification of chemical groups, including amino, carboxylic acid and phenol groups (Li et al., 2010), which can serve as linkers for drug decoration. Animal studies suggest that phage can carry drugs to its sidewall, penetrate the blood barrier, and then deliver the drug to brain (Carrera et al., 2004).

Previous studies have revealed that peptides can be conjugated to doxorubicin with NHS and EDC (Arap et al., 1998). Obviously, pVIII coat protein of phage with ∼2700 copies can also be decorated with drugs. In order to observe the visualization of phage infection, NHS chemistry was utilized for chemical conjugation of biotins on the phage particle to form biotinylated phages (BIO-phages) (Nakamura et al., 2001; Nakamura et al., 2002), which can be detected under confocal fluorescence microscopy by Biotin-Avidin-System (BAS). The first report on filamentous phage drug carrier is about the targeting eradication of bacteria, in which a large load of chloramphenicol (about 3000 molecules/phage) was linked to the lysines of phage on pVIII and delivered into the target cells by IgG-binding ZZ domain which was also displayed on pIII (Yacoby et al., 2006). In order to increase the loading capacity of filamentous phage, the phage coat carboxyl residues instead of the amine residues are used to conjugate chloramphenicol by EDC chemistry with over 40000 chloramphenicol molecules/phage (Yacoby et al., 2007), and thus leading to complete growth inhibition toward pathogens.

Besides antibacterial nanomedicines, the drug-carrying phage can also be applied for antitumor therapy. In the proof of concept study, the antibody-targeted phage is modified with cytotoxic drugs by a covalent bond or a cathepsin-B cleavable peptide, and then treated specific cancer cells. The results indicated that the potency of selective cell killing was significantly enhanced with a factor of >1000 over the corresponding free drugs (Bar et al., 2008).

Moreover, polymers which can load drugs and protect them from degradation are another good alternative for the conjugation of drugs with phage particles. For example, the FA-M13-PCL-P2VP nanoassemblies were developed, which composed of two main functional modules: one is M13 phage modified with folic acid constitute the shell acting as targeting moieties and drug carriers; the other is the PCL-P2VP copolymer loaded with doxorubicin constitute the core and used for drug protection and release. The results showed that the DOX-loaded particles also had a significantly higher tumor uptake and selectivity compared to free DOX (Suthiwangcharoen et al., 2011). Without release, some therapeutics just needed to be delivered to the target site, such as photosensitizer (PPa) and radionuclides. Through decorating with PPa on the surface via EDC and displaying with SKBR-3 cell-binding peptide (VSSTQDFP), Mao’s group prepared the p-PPPa complexes using for the selective killing of SKBR-3 cancer cells (Gandra et al., 2013a).

### 4.3 Phage-liposome complex for drug and gene delivery

Liposomes as artificial phospholipid vesicles have been developed as pharmaceutical carriers with biomedical profiles, such as good biocompatibility, little or no side effect, easy biodegradation, and large loading capacity (Torchilin, 2005; ElBayoumi & Torchilin, 2010). In order to optimize the characterization of liposomes, many new liposome formulations have been produced for longer half-life and targeting delivery. In fact, bacteriophages may be the good complementary to liposomes.

Recently, phage particles have been combined with liposomes to form new phage-liposome complexes. For instance, by multivalent electrostatic interactions, cationic liposomes are assembled on the surface of M13 phage displayed with negative charged peptides to form phage-liposome complexes (Ngweniform et al., 2009), which not only stabilize the liposome but also help ZnPC-loaded liposomes arrive specific cells. This provides a novel approach for delivering liposomes to desired targets. Through altering the ratio of liposome/phage, the structure evolution of phage-liposome complexes was studied, including “beads-on-rod” structure, nanoweb structure, short rings or spirals and phage matrix embedded with liposomes (Kalarical Janardhanan et al., 2010). Based on the targeting ability of engineered phage and loading capacity of liposomes for anticancer drugs, the nanoweb phage-liposome complexes are characterized and used as an efficient novel vehicle for drug (ZnNC) delivery. Compared with simple liposomes, nanoweb complexes can deliver more drugs to targeted cancer cells and result in increased death of cells.

## 5. Phage-mimetic nanoparticles-mediated delivery

### 5.1 Integrating targeting peptide/protein into liposome for targeted drug and gene delivery

With the excellent profiles of easy synthesis, good biocompatibility, flexible surface modification, low toxicity, and large drug/gene loading capacity, liposome as the first choice of gene and drug carrier has been used successfully in clinical trials (Noble et al., 2014). However, with the progress of liposome, quick clearance from circulation and nonspecific targeting has been the new stumbling blocks in liposome delivery. So some methods are developed and applied for liposome surface modification. Due to lower cost and facile chemical modifications, different length and density PEG was utilized to modify liposomes (Fang et al., 2005; Dos Santos et al., 2007; Maldiney et al., 2011). Although more attention has been paid to PEG coating and much progress was achieved, biomimetic coating was still adopted. One significant example is the short peptides that screened from phage libraries and used for targeting. By direct chemical conjugation, synthesized peptides were incorporated into liposomes for cell targeting in monomeric form (Pastorino et al., 2006; Stefanick et al., 2013; Noble et al., 2014) or multivalent form (Accardo et al., 2013; Gray et al., 2013; Avvakumova et al., 2014). In addition, peptides with different function were inserted into a same liposome to endow the complex more versatile. For example, rMSC-targeting peptide and a NLS peptide were encapsulated into a liposome protamine/DNA lipoplex (LPD), which improved its targeting capacity to rMSC and nuclei (Ma et al., 2013). These LPD nanoparticles were also used to deliver eye-specific genes to eyes for improving the vision of blind mice *in vivo* (Rajala et al., 2014).

However, the cost of synthesized peptides and the reproducibility of these systems are still major challenges for pharmaceutical applications. More importantly, the chemical modification of synthesized peptides makes the preparation process more complicated, even possibly alter the property and specificity of peptides (Emerich & Thanos, 2008). Therefore, a new alternative need to be recommended. Because of the “membranophilic” nature of phage major coat proteins, numerous studies have showed that the “wild-type” phage protein can be integrated into micelles and phospholipid bilayers (Stopar et al., 2003; Jayanna et al., 2009). So phage fusion pVIII coat proteins are directly inserted into liposomes and formed a new phage protein-liposome nanovehicles, which have been used in several drug and gene delivery (Bedi et al., 2011; Petrenko & Jayanna, 2014).

### 5.2. Transferring phage protein/peptide to nanoparticles for targeted drug and gene delivery

To date, a large number of nanoparticles are considered as potential drug and gene delivery carriers, such as polymeric nanoparticles (Nicolas et al., 2013), metal nanoparticles (Conde et al., 2012), dendrimers (Cheng et al., 2011), exosomes (Kooijmans et al., 2012) and so on. However, the deliver efficiency of these nanoparticles is too low due to nonspecific targeting ability. Therefore, many targeting peptides are conjugated on their surface to improve specific targeting (has been reviewed elsewhere (Pearce et al., 2012; Levine et al., 2013)) . Through transferring mesenchymal stem cells (MSCs)-targeting pVIII from phage to virus-mimetic magnetic silica nanoclusters (VMSNCs), the VMSNCs loaded with interesting genes can be targeting delivered to MSCs at a higher efficiency than commercially available vectors (Gandra et al., 2013b).

Beyond that, phage proteins themselves can assemble into nanoparticles for gene delivery. After inserting with specific targeting peptides, the phage will produce fusion pVIII (fpVIII) which has new targeting ability. Based on this, MCF-7 cells-targeting pVIII proteins self-assemble with polymeric PEG-PE to form phage-micelles, which have been used for targeting delivery of poorly soluble drugs (Wang et al., 2010; Vladimir, 2012). Similarly, Deepa Bedi et al. (2013) utilize fpVIII to encapsulate siRNA and form a kind of phage-mimetic nanoparticle, named “nanophage”, which can deliver siRNA to the target region and result in gene silencing.

## 6. *In vivo* applications of phage and phage-mimetic nanoparticles

Since the report of direct intralesional injection of bacteriophage in 6 patients with staphylococcal boils by Bruynhoge R and Maisin J in 1921, a large quantity of clinical applications of phage have been performed and reviewed elsewhere (Debattista, 2004; Fischetti et al., 2006; Kropinski, 2006; Skurnik & Strauch, 2006), most of them are mainly focus on antibacterial therapies. Subsequently, with the discovery of phage display technology by George P. Smith, many targeting peptides are explored from animals or patients *in vivo* using phage libraries. In 1996, the first *in vivo* selection of organ targeting peptide was selected (Pasqualini & Ruoslahti, 1996), and then a wide variety of organ and disease tissues were applied for targeting peptides exploration, including brain, kidney, lungs, liver and so on (Bábíčková et al., 2013). The first phage-mediated target gene transfer *in vivo* was reported in 2002 by Mechael A. Burg et al. (2002). Thereafter, by combining with camptothecin (CPT), EGF-targeted phagemid vector-mediated gene transfer efficiency was significantly improved up to 45% and its transduction *in vivo* was also assessed in PC-3 tumor xenografts of mice. Meanwhile, Frenkel and Solomon (2002) demonstrated that filamentous phage which delivered antibody to the brain could penetrate into the central nervous system in an intact form. Recently, it was reported that FDA-approved phase I clinical trial showed no safety concern (Chanishvili, 2012). In a word, all those above experiments show that no significant side effects are appeared in animals or humans *in vivo* after treated with phages.

During last several decades, a large quantity of administration methods have been utilized for phage delivery *in vivo*. One of the most common administration methods is intravenously delivery by directly injecting phage particles into blood. Phages are directly contacted with circulating blood first and then arrived different areas of the body. So this method is generally used for selection of peptides targeting vascular receptors of organs (Pasqualini & Ruoslahti, 1996; Arap et al., 2002; Yao et al., 2005; Jung et al., 2012) or tumor *in vivo* (Newton et al., 2006; Larimer & Deutscher, 2014). Because of the blood-brain barrier, intranasally delivered phage is exploited for targeting central nervous system (CNS) of brain (Frenkel & Solomon, 2002; Rakover et al., 2010; Lochhead & Thorne, 2012). Additionally, transdermal deliver (Prausnitz & Langer, 2008), intestinal delivery, oral administration, and intraperitoneal injection (Akita et al., 2006) are also used for *in vivo* delivery of phage and phage-mimetic nanoparticles. The closer administration to target organ or tissue, the stronger affinity will be obtained (Bábíčková et al., 2013).

Even a lack of reports on adverse effects, it does not mean that there is no any side effect of phage in animal and human therapy. After entering into animals or humans, bacteriophages can interact with host immune system including the phage immunogenicity and the immune-modulatory of phage (Górski et al., 2012). These interacts can induce specific immune response, and trigger innate or adaptive immune responses, such as phagocytosis, cytokines production and nonspecific antibodies production. All of these reactions may impact the effects of phage therapy *in vivo*. Obviously, as one kind of nanoscale biomaterial, phages need to be considered as nanoparticles for investigating their operation mechanism *in vivo* (Henein, 2013).

## 7. Conclusion

Filamentous bacteriophage has been exploited in the development of target drug delivery as virus-based delivery system. Phage enables target-selective delivery in several pathways: Firstly, the phage displayed with the target-specific peptides or antibodies can be used as nanocarriers of chemical drugs or gene drugs. Secondly, the phage displayed with target-specific peptides or antibodies can be conjugated with other vehicles (such as liposomes, inorganic nanoparticles) to form a novel delivery system. Third, the peptides or antibodies selected from a random phage library can be directly used as raw material to build new delivery systems by themselves or combining with other vehicles.

Though progress on filamentous phage-based delivery system has been made during last decades, the potential of this biomaterial needs further exploitation. Lately, the new concept of “self-navigating nanomedicines” based on filamentous bacteriophage was first proposed on the Tech Connect World Innovation Conference and Expo Techconnect Briefs 2017 (Petrenko & Gillespie, 2017), which may direct the future development of phage-based drug delivery system. As the research moves along, we believe that filamentous phage and phage-mimetic nanoparticles will play a crucial role for the development of precise and personal medicine.
